# Attitudes Toward and Familiarity With Virtual Reality Therapy Among Practicing Cognitive Behavior Therapists: A Cross-Sectional Survey Study in the Era of Consumer VR Platforms

**DOI:** 10.3389/fpsyg.2019.00176

**Published:** 2019-02-08

**Authors:** Philip Lindner, Alexander Miloff, Elin Zetterlund, Lena Reuterskiöld, Gerhard Andersson, Per Carlbring

**Affiliations:** ^1^Department of Psychology, Stockholm University, Stockholm, Sweden; ^2^Center for Psychiatry Research, Department of Clinical Neuroscience, Karolinska Institutet, Stockholm, Sweden; ^3^Stockholm Health Care Services, Stockholm County Council, Stockholm, Sweden; ^4^Department of Behavioral Sciences and Learning, Linköping University, Linköping, Sweden; ^5^Department of Psychology, University of Southern Denmark, Odense, Denmark

**Keywords:** virtual reality, therapist, cognitive behavior therapy, dissemination and implementation, eHealth

## Abstract

Virtual reality exposure therapy (VRET) is an efficacious treatment for fear and anxiety and has the potential to solve both logistic issues for therapists and be used for scalable self-help interventions. However, VRET has yet to see large-scale implementation in clinical settings or as a consumer product, and past research suggests that while therapists may acknowledge the many advantages of VRET, they view the technology as technically inaccessible and expensive. We reasoned that after the 2016 release of several consumer virtual reality (VR) platforms and associated public acquaintance with VR, therapists’ concerns about VRET may have evolved. The present study surveyed attitudes toward and familiarity with VR and VRET among practicing cognitive behavior therapists (*n* = 185) attending a conference. Results showed that therapists had an overall positive attitude toward VRET (pros rated higher than cons) and viewed VR as applicable to conditions other than anxiety. Unlike in earlier research, high financial costs and technical difficulties were no longer top-rated negative aspects. Average negative attitude was a larger negative predictor of self-rated likelihood of future use than positive attitude was a positive predictor and partially mediated the positive association between VRET knowledge and likelihood of future use, suggesting that promotional efforts should focus on addressing concerns. We conclude that therapist’s attitudes toward VRET appear to have evolved in recent years, and no longer appear to constitute a major barrier to implementing the next generation of VR technology in regular clinical practice.

## Introduction

Anxiety disorders are characterized by excessive fear of specific situations and/or stimuli, as well as anticipatory anxiety and dysfunctional, fear-reinforcing avoidant behaviors. Lifetime prevalence is slightly above 30% (Kessler et al., 2005, 2012), making it one of the most common mental health problems. Exposure therapy, delivered as part of a cognitive behavior therapy (CBT) intervention or even as stand-alone treatment, is highly effective in reducing fear and anxiety (Norton and Price, 2007). Providing and conducting traditional, *in vivo* exposure therapy is, however, associated with some limitations and logistic challenges. The median delay between onset to treatment seeking can be as high as 20 years or more for some anxiety disorders (Wang et al., 2005; Raven et al., 2017) and drop-out during face-to-face exposure therapy is not uncommon, although rates are not higher than for other psychological treatments (Ong et al., 2016). There is thus a need for novel interventions that supplement traditional exposure therapy, preferably in the form of accessible interventions, delivered either as part of a stepped-care model (Nordgreen et al., 2016) or by targeting at-risk individuals with disabling fear and anxiety who are disinclined or perceive barriers to seek traditional treatment (Issakidis and Andrews, 2002; Dezetter et al., 2015). As to issues in conducting *in vivo* exposure therapy, exposure stimuli may be inaccessible (e.g., in cases of fear of thunderstorms or flying), difficult to acquire and maintain (e.g., in cases of insect phobia), or to control (e.g., in public speaking phobia).

Virtual reality (VR) technology promises to address some of these challenges (Botella et al., 2017). In short, VR refers to technology that creates the experience of presence in a computer-generated environment by presenting an interactive, three-dimensional simulation of a virtual physical world, while at the same time withholding sensory input from the real world. Most commonly, this is achieved through the use of a head-mounted display (HMD) that covers the eyes and simulates stereoscopic depth perception by presenting slightly different views of the virtual world to each eye, and by using sensors to track head motion to simulate looking around the virtual environment (Freeman et al., 2017). In its simplest form, populating the virtual environment with realistically rendered fear-inducing stimuli is enough to conduct VR exposure therapy (VRET), although the total control over the virtual environment also enables presentation of stimuli, contexts, and tasks not possible in *in vivo* exposure therapy (Lindner et al., 2017). This includes gamified tasks to promote engagement or creating engaging self-help platforms featuring virtual therapists (Miloff et al., 2016). Research into using VR to conduct exposure therapy began in the 1990s and there are now over 30 randomized controlled trials revealing high efficacy and effect sizes comparable to *in vivo* exposure therapy (Powers and Emmelkamp, 2008; Opriş et al., 2012; Carl et al., 2018; Fodor et al., 2018). Crucially, treatment improvements are also observed on behavioral measures, revealing that VR exposure translates into reduced fear also of real-world stimuli (Morina et al., 2015).

Despite proven efficacy and potential to solve many of the practical issues of conducting *in vivo* exposure therapy, VRET has yet to spread beyond specialized clinics and research settings, with no widespread implementation in ordinary clinical settings (Gega, 2017) and very limited translational efforts aimed at developing efficacious self-help applications. This is likely the result of both technological and human factors. Past generations of VR hardware were expensive (costing as much as 10,000 USD), inaccessible, physically heavy, and required a high degree of technical proficiency to program and use. Additionally, graphical resolution, field-of-view, and refresh rates were low, even by the display standards of the time, limiting the immersive experience and increasing the risk of inducing motion sickness among users (Rebenitsch and Owen, 2016). Since VRET has thus far remained a niche research area, there has been little research on implementation in regular care and very little research on how ordinary therapists view VRET. Research from 2010–2012 suggested that although therapists had an overall positive view of VRET (pros outweighing cons), there were also concern about required training, technical difficulties in operating, financial costs in acquiring, low immersion, and low efficacy, as well as a general unfamiliarity with the technology (Kramer et al., 2010; Segal et al., 2011; Schwartzman et al., 2012).

**FIGURE 1:**
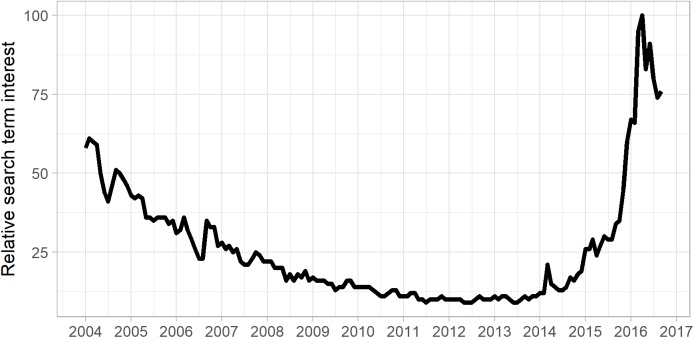
Increased public interest in VR over time as revealed by Google searches. Relative interest for search term “Virtual Reality” during the period 2004/01–2016/09 (time of survey data collection), extracted through the Google Trends platform.

The unprecedented and rapid development of consumer VR technology in recent years, culminating in the 2016 highly publicized release of several commercial VR hardware platforms intended for the consumer market (e.g., the Oculus Rift, HTC Vive, Samsung Gear VR, Playstation VR, and Google Daydream) promises a paradigm shift in the design and availability of VR mental health interventions. High-end, smartphone-based, or stand-alone HMDs now cost as little as 100 USD and there are well-established channels for application development (game engines) and distribution (digital marketplaces). For these reasons, many of the past findings on how therapists and clients view VRET may no longer hold true. In particular, many of the barriers identified in past research (e.g., high cost of equipment, technical difficulties, and low graphical quality) have been remedied with the release of consumer VR hardware platforms (Lindner et al., 2018). The release of these platforms has likely fueled increased public familiarity with VR technology, as indicated by a recent increase in the relative popularity of the Google search term “VR” (see Figure 1). However, many therapists may be unaware of the recent advances in VR technology and its availability. Familiarity with and experience of using VR technology may thus be an important factor in explaining therapists’ attitudes toward VR therapy and along with pros/cons of VR treatment, may indicate whether they would consider using the technology in their own practice or recommending it to their patients.

To investigate whether earlier research on therapists’ attitudes toward VR therapy still hold true in the era of consumer VR platforms, and what barriers to implementation that remain today, and whether attitudes, familiarity with VR technology, and therapy have an impact on these perceived likelihood of future use of VR in clinical practice, we conducted a survey study on the subject among practicing cognitive behavior therapists.

## Materials and Methods

### Ethics Statement

According to Swedish legislation (2003:460), no ethical approval is required for research (like the current study) that does not involve collecting data traceable to a specific individual, or sensitive data (defined according to 1998:204, e.g., ethnicity, religious, or political views, health and sexuality), or intends, or risks, having a physically or psychologically effect on participants. Participants were informed that by responding to the survey, they consented to the terms described in the first part of the survey. The ethics and consent statement, along with the rest of the survey, is included in the Supplementary Material.

### Participants and Procedure

Participants (*n* = 185) were recruited among visitors to the 46th congress of the European Association of Behavioral and Cognitive Therapies (August 31st to September 3rd, 2016), in Stockholm, Sweden, with the sole inclusion criteria of practicing CBT to some degree. Participants were approached at the congress venue by one of four surveyors and asked to participate in a study on therapists’ views on and experience of using VR in therapy. All participants were shown a VR headset, but no standardized operational definition of VR or VRET was provided in order not to introduce any bias. If participants remained ignorant of what VR refers to, they were given a brief, non-standardized description of the how the technology works. Participation was compensated immediately with a scratch lottery ticket worth 30 SEK (approximately 3.50 USD). Based on a total conference attendance number of *n* = 1858, the theoretical response rate was 10%. Due to the nature of data collection, however, it was not feasible to accurately record the number of approached individuals who declined to participate. Participants were primarily psychologists (*n* = 147; 79.5%), female (*n* = 125; 67.6%), 41.98 (*SD* = 12.69) years old and with 11.71 (*SD* = 10.66) years of clinical experience on average. See Table 1 for additional sample characteristics. The conference was organized by the two prominent CBT member organizations in Sweden, and as revealed by the relatively high percentage of participants stating that they only work clinically, the conference attracted a large number of clinicians not otherwise involved in research, in particular Swedish clinicians. Participants’ country of residence was not recorded due to traceability reasons (see section “Ethics Statement”) and since for analysis purposes, countries would have to be collapsed into regions, the choice of which would be arbitrary.

**Table 1:** Sample characteristics.

Variable	*N* (%) or mean (*SD*)
Average age	41.98 (12.69)
**Training background**	
Psychologist	147 (79.5%)
Psychiatrist	13 (7.0%)
Social worker	8 (4.3%)
Nurse	7 (3.8%)
Counselor	6 (3.2%)
Other	4 (2.2%)
Average years as CBT practitioner (SD)	11.71 (10.7)
**^∗^Time spent between clinical work and research**	
Only clinical work	52 (28.1%)
Both clinical work and research	133 (71.9%)
**Works clinically with following disorder (multiple answers)**	
Anxiety disorders	160 (86.5%)
Family and couples therapy	14 (7.6%)
Disruptive behavior disorders	27 (14.6%)
Eating disorders	33 (17.8%)
Gambling disorder	8 (4.3%)
Mood disorders	131 (70.9%)
Neuropsychiatric disorders (ADHD and autism)	38 (20.5%)
Personality disorders	50 (27.0%)
Psychotic disorders	13 (7.0%)
Psychosomatic disorders	45 (24.3%)
Substance use disorders	17 (9.2%)
Other disorders	27 (14.6%)

^∗^*Original response format 0–10, with anchors “Only research/other” and “Only clinical.” Binarized for descriptive purposes due to bimodal distribution and reverse-scored for regression models.*

### Survey

The survey, which was exclusively in English, could be filled out either on paper (*n* = 133; 71.9%) or online (*n* = 52; 28.1%) and was subdivided into three sections. The first page included information about the aims of the study, the research team behind the study, compensation offered, that participation was anonymous, that data collection was in accordance with Swedish ethical standards, and that by responding to the survey, they consented to these terms. Unlike past research (Segal et al., 2011), we chose to focus (albeit not exclusively) on using VR to conduct exposure therapy, since this is by far the most researched and widespread application of VR technology in a therapeutic setting (Turner and Casey, 2014; Valmaggia et al., 2016; Freeman et al., 2017). Additionally, by conducting the survey in a sample of participants visiting a CBT congress, we likely also excluded therapists with other primary theoretical orientations (e.g., psychodynamic), in line with our choice to focus on VRET. Missing data were not permitted in the online survey version and were negligible in the paper version, at most *n* = 8 for any question (participant age) and *n* = 2 for outcomes; hence, missing data were omitted case-wise.

The first survey section included questions on demographics, professional background, and clinical focus. The second section included items on self-reported familiarity with VR technology and knowledge of the extant literature on VRET efficacy (response format: score 0–10 with anchors “Not at all” and “Very familiar”), and experiences of using VR in both therapeutic and non-therapeutic settings (original response format: “No,” “Yes, on occasion,” or “Yes, frequently”). Participants also indicated the likelihood of future use of VR (response format: score 0–10 with anchors “Not at all” and “Definitively”), and which types of mental health problems (diagnostic categories) they think VR can be used with.

**FIGURE 2:**
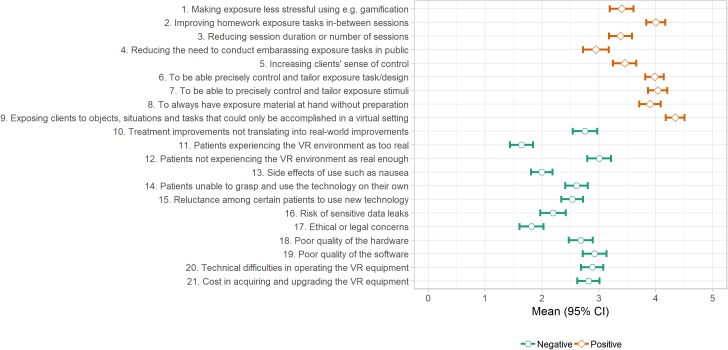
Therapist ratings of positive and negative aspects of VRET.

Finally, in the third section, respondents rated how positive or negative (respectively) they viewed nine potential positive and 12 potential negative aspects of VRET using a six-step (0–5) rating scale with verbal anchors at zero (“Matters not” or “No concern”) and five (“Very positive” or “Very negative”). See Figure 2 for included items. Of note, in past research (Segal et al., 2011), ratings were made using six-step scale scored 1–6, entailing a systematic mean difference of +1 between the current study and past research. Since the aim of the current study was not to directly replicate past findings, the item pool was partly drawn from past research (Segal et al., 2011; Schwartzman et al., 2012), yet revised and supplemented collaboratively by the authors in light of recent technological advancements. This included items covering novel aspects such as in-between session homework and use of gamification. In order to collect standardized survey data with low-threshold responding, the item pool was not designed to be exhaustive and the survey did not allow for additional free-text options to be supplied. A unidirectional item response format with explicit positive/negative definitions, rather than a generic positive-to-negative bidirectional response format, was chosen to emphasize the impact direction. Cronbach’s alpha for the negative items were 0.84 (95% CI: 0.8–0.87) and for the positive items 0.87 (95% CI: 0.85–0.90), revealing mean scores to be appropriate.

### Analyses

Analyses were conducted in the R (version 3.4.2) statistical environment. As in previous research (Segal et al., 2011; Schwartzman et al., 2012), we calculated descriptive statistics for ratings of each VRET aspect, as well as average positive and average negative aspect scores. Because we hypothesized that clinicians working with a specific disorder would be more inclined to view VR as applicable in treating this disorder, we calculated frequencies of positive answers in both the whole sample and the two groups (those who work, or do not work with the disorder in question), using Fisher’s exact test to compare frequencies. To explore which factors were the most important in predicting self-rated likelihood of future use of VR in clinical settings, we calculated a single multiple regression model that included all hypothesized predictors: non-clinical experience of using VR technology (dummy coded to either 0 or 1, no or yes, due to low frequency of “Yes, frequently” responses), familiarity with VR technology, knowledge of VRET, average positive rating, average negative rating, awareness of recent VR technology releases, and time split between clinical work and research/other (rated 0–10, with zero being only clinical work). Finally, based on the multiple regression results and a theorized working model (see below), mediation analyses were conducted within a structural equation modeling framework, using the *lavaan* R package (Rosseel, 2012), simultaneously testing (with robust standard errors) whether there in addition to a direct path between the primary predictor and the outcome (path C), there would also be statistically significant paths between the primary predictor and the mediator (path A), and between the mediator and the outcome (path B), such that the product of these two paths (A^∗^B) would constitute an indirect, mediating path. Partial mediation was defined as a significant indirect path in addition to a surviving direct path. Power for the indirect effect of a specific model was determined *post hoc* using Monte Carlo simulations (10,000 replications and 20,000 draws; Schoemann et al., 2017).

## Results

### Familiarity With VR Technology and Knowledge of VRET

The absolute majority (*n* = 158; 86.34%) of participants reported no experience of using VR in a clinical setting, and only *n* = 6 (3.28%) reported frequent use, with the remaining *n* = 19 (10.39%) reporting occasional use (*n* = 2 missing). A larger proportion of respondents (*n* = 65; 35.52%) reported using VR frequently or occasionally in another setting (e.g., gaming). Approximately half of participants (*n* = 96; 52.17%) reported being aware of there now being consumer VR platforms on the market. Average familiarity with VR technology was low, with a mean of 4.05 (*SD* = 2.97), as was knowledge of VRET (*M* = 3.37, *SD* = 2.86), both rated on a 0–10 scale. Nearly a quarter of participants (*n* = 42; 22.83%) reported no knowledge of VRET at all.

### Views of VRET

Top-rated positive aspects of VRET (rated 0–5) included enabling exposure to objects, situations, and tasks that can only be done in a virtual setting, being able to precisely control and tailor the exposure stimuli, and task/design, improving homework exposure tasks between sessions, and to always have exposure material at hand. Patients not experiencing the VR environment as real enough were the highest rated negative aspect, along with poor quality of software. See Figure 2 for full results. Average positive score was significantly higher than average negative score [Δ*M* = 1.23 (95% CI: 1.08–1.37), *t* = 16.62, *p* < 0.001]. See Figure 4A for correlation matrix.

When asked what types of mental health problems they believe VR could be used with, almost all participants (96.74%) selected anxiety disorders, followed by mood disorders (52.72%), neuropsychiatric disorders (45.65%), eating disorders (44.57%), gambling disorder (42.39%), and substance use disorders (40.22%). Psychosomatic disorders (29.35%), disruptive behavior disorders (26.63%), psychotic disorders (20.65%), personality disorders (18.48%), family and couples therapy (10.33%), and other disorders (7.61%) were less popular alternatives. However, when subdividing the sample based on whether the respondent reported working with the specific disorder or not, in all cases, respondents who worked with a specific disorder were more likely to view VR as applicable, although the difference was not statistically significant in all cases (see Table 2).

**Table 2:** Positive responses to what types of mental health problems VR can be used with.

Types of mental health problems that VR can be used with	Positive responses among therapists who work with the disorder: *n* (%)	Positive responses among therapists who do not work with the disorder: *n* (%)	Fisher exact *p*-value	Positive responses in total: *n* (%)
Anxiety disorders	155 (97.5%)	23 (92.00%)	*p* = 0.19	178 (96.7%)
Family and couples therapy	3 (21.4%)	16 (9.41%)	*p* = 0.16	19 (10.3%)
Disruptive behavior disorders	10 (37.0%)	39 (24.84%)	*p* = 0.24	49 (26.6%)
Eating disorders	20 (60.6%)	62 (41.06%)	*p* = 0.053	82 (44.6%)
Gambling disorder	5 (62.5%)	73 (41.48%)	*p* = 0.29	78 (42.4%)
Mood disorders	72 (55.4%)	25 (46.30%)	*p* = 0.33	97 (52.7%)
Neuropsychiatric disorders (ADHD and autism)	24 (63.2%)	60 (41.10%)	*p* = 0.02	84 (45.7%)
Personality disorders	15 (30.0%)	19 (14.18%)	*p* = 0.02	34 (18.5%)
Psychotic disorders	7 (53.9%)	31 (18.13%)	*p* = 0.01	38 (20.7%)
Psychosomatic disorders	21 (46.7%)	33 (23.74%)	*p* = 0.005	54 (29.4%)
Substance use disorders	12 (70.6%)	62 (37.13%)	*p* = 0.01	74 (40.2%)
Other disorders	9 (33.3%)	5 (3.18%)	*p* < 0.001	14 (7.6%)

### Factors Associated With Likelihood of Future Use

In a single multiple regression model predicting self-rated likelihood of future use, non-clinical experience of using VR technology (*B* = 1.07, *SE* = 0.39, *p* = 0.007), knowledge of VRET (*B* = 0.51, *SE* = 0.09, *p* < 0.001) and average positive rating (*B* = 0.49, *SE* = 0.20, *p* = 0.017) were positively associated with greater likelihood of future use. Average negative rating was negatively associated with likelihood (*B* = -0.86, *SE* = 0.20, *p* < 0.001). There was no association with awareness of recent VR technology releases (*p* = 0.24), time split between clinical work and research/other (*p* = 0.06), or familiarity with VR technology (*p* = 0.07).

**FIGURE 3:**
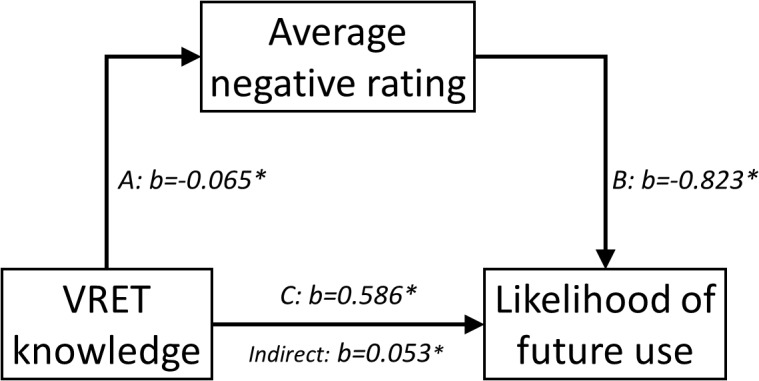
Mediation model.

**FIGURE 4:**
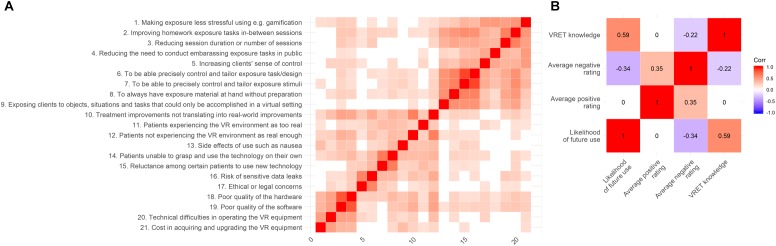
Correlation matrices of **(A)** positive and negative aspects, and **(B)** numeric variables used in the mediation models.

Based on these findings, mediation analyses were conducted to explore whether the effects of VRET knowledge and non-clinical experience of VR (primary predictors, respectively) on likelihood of future use (outcomes) were partially mediated by indirect effects via average positive and negative ratings (mediators, respectively), i.e., two-by-two totaling four separate models. These analyses were based on a working model, distilled from the literature examining implementation of prolonged exposure for veteran post-traumatic stress disorder (Becker et al., 2004), hypothesizing that therapist’s personal views of VRET (in particular negative ones), rather than knowledge of efficacy or personal experience *per se* (which are likely necessary but not sufficient conditions), are the prime drivers of implementation potential, although the latter may influence the former. Four separate models were run to explore all possible combinations of hypothesized relations, making no assumptions about non-relevant paths as would be required for a single model. Results revealed that average negative rating partially mediated the association between knowledge of VRET and likelihood of future use (*p* = 0.014); see Figure 3. Using Monte Carlo simulations, power was estimated to 82% for this indirect effect. A near-significant indirect path (*p* = 0.066) between non-clinical experience and likelihood of future use through average negative rating was also found. No such indirect effects were found for average positive ratings. See Table 3 for full results. See Figure 4B for correlation matrix of variables.

**Table 3:** Mediation results.

	Mediator: average positive rating			Mediator: average negative rating		
Predictor: non-clinical experience	*B*	*SE*	*p*	*B*	*SE*	*P*
Path A: mediator ∼ predictor	0.047	0.141	0.737	-0.277	0.129	0.031
Path B: outcome ∼ mediator	0.227	0.258	0.378	-1.062	0.238	<0.001
Path C: outcome ∼ predictor	2.226	0.490	<0.001	1.942	0.466	<0.001
Indirect effects (A^∗^B)	0.011	0.035	0.760	0.294	0.160	0.066
Total effects	2.237	0.490	<0.001	2.237	0.490	<0.001

**Predictor: VRET knowledge**	***B***	***SE***	***p***	***B***	***SE***	***P***

Path A: mediator ∼ predictor	-0.004	0.022	0.843	-0.065	0.022	0.003
Path B: outcome ∼ mediator	0.301	0.241	0.211	-0.823	0.230	<0.001
Path C: outcome ∼ predictor	0.641	0.083	<0.001	0.586	0.081	<0.001
Indirect effects (A^∗^B)	-0.001	0.007	0.844	0.053	0.022	0.014
Total effects	0.640	0.084	<0.001	0.640	0.084	<0.001

## Discussion

Despite past findings demonstrating high efficacy of VRET, and positive attitudes among clinicians toward using this technology in clinical practice, VRET has not yet seen widespread implementation in routine care. This is likely due to both technological and human barriers, yet while the former have largely been addressed with the recent release of consumer VR platforms, changes of the latter were unknown. The rationale for the current study was to survey, for the first time in the era of consumer VR platforms, attitudes toward, familiarity with, and knowledge of VRET among practicing CBT clinicians. Our results are in line with past findings that clinicians overall have a positive attitude toward VRET, and show for the first time that clinicians view VR as applicable not only in conducting exposure therapy to treat anxiety disorders, but also a range of other disorder types. Negative attitudes were a larger negative predictor of self-rated likelihood of future use than positive attitudes were a positive predictor, and partially mediated the positive associations between VRET knowledge and (near-significantly so) non-clinical experience of using VR on likelihood of future use.

These findings suggest that in implementation and dissemination efforts, clinicians’ concerns over perceived negative aspects of VR treatments need to be addressed, while specifically promoting positive aspects (e.g., providing exposure paradigms not possible in real life) may be of lesser importance since these are likely self-apparent to practicing clinicians. The stronger influence of negative over positive aspects is a ubiquitous psychological phenomenon (Baumeister et al., 2001) and with regard to the implementation of VR in clinical contexts, historical parallels may be drawn to the dissemination of a related psychological treatment, traditional exposure therapy. Clinical implementation has proven challenging (Gunter and Whittal, 2010), in part because of negative preconceptions among clinicians, which impacts treatment availability and even quality of delivery (Farrell et al., 2013): for example, many clinicians hold (erroneous) beliefs that exposure therapy will likely harm their patients and that manualized treatments fail to address patients’ idiosyncratic needs or will be detrimental to the therapeutic alliance, along with (erroneous) beliefs that results from randomized controlled trials offer little guidance for clinical practice due to their samples being non-representative (Gunter and Whittal, 2010). In our study on view of VR therapy, two of the highest rated concerns were improvements not translating into real-world improvements and patients not experiencing the virtual environment as real enough, both of which are therapeutic rather than technical concerns. Based on the extant literature, there is no empirical support for any of these concerns (Ling et al., 2014; Morina et al., 2015), yet the challenging dissemination of traditional exposure therapy highlights the need to nonetheless address these concerns when disseminating VR treatments. Standardized training programs may be needed to increase knowledge of VRET and improve attitudes toward it, as has been the case in disseminating traditional exposure therapy (Harned et al., 2013, 2014).

Such training programs should include the technical skills required to conduct VR treatment. The perceived need for technical training, and willingness to even participate, was not explicitly covered by the current study and should be addressed in future research. Although specific concerns about technical aspects of VR treatment were rated lower than in previous research conducted before the advent of consumer VR platforms (Segal et al., 2011), we found that such concerns continue to be among the highest ranked. It is likely that a low rate of familiarity with VR technology explains these findings: only close to a third of our sample reported having used VR technology in non-clinical settings, a factor which was associated with a greater likelihood of future use, even when controlling for other factors such as knowledge of VRET efficacy. Although causal inferences cannot be made from cross-sectional data, these findings do suggest that in time, as VR becomes part of everyday technology use, it will become more natural for clinicians to use it to conduct psychological treatments. The 2016 release of several consumer VR platforms such as the Oculus Rift, HTC Vive, Playstation VR, Samsung Gear VR, and more constitutes a paradigm shift in the availability of VR technology and the possibilities to implement VRET in regular care settings (Powers and Carlbring, 2016). Mere availability of technology is, however, seldom a sufficient condition for its dissemination. Our findings may beneficially be used to guide experimental research on development aspects of VRET applications: user-friendliness in particular, will need to be high, preferably on par with that of other consumer (e.g., smartphone) applications, or at least that of other medical technology. Future research should examine in detail clinicians’ perceived structural barriers to, and facilitators of, implementation of VR in clinical settings.

Almost all participants (96.74%) indicated that VR could be used in the treatment of anxiety disorders. This is unsurprising given that clinical applications of VR technology have largely been focused on conducting exposure therapy (Freeman et al., 2017). Interestingly, therapists who indicated that they worked clinically with a specific disorder type were more inclined to perceive VR as applicable in treating that specific disorder, although the difference was not significant for all disorder types. This suggests that therapists with specific area expertise see clinical applications of the technology beyond exposure therapy, e.g., in treating eating disorders, neuropsychiatric disorders, personality disorders, and psychotic disorders. Applicability ratings were also high for psychosomatic disorders, gambling disorder, and substance use disorders, popular treatments of which often include exposure elements (e.g., Ljótsson et al., 2014; Hedman et al., 2016). Future research should thus explore the efficacy of VRET in treating not only anxiety disorders, but also disorders characterized by aversive bodily experiences or appetitive conditioning. Research shows promise in areas such as stress (Baños et al., 2009; Serrano et al., 2016), eating disorders (Perpina et al., 2003; Gorini et al., 2010), pain relief (Hoffman et al., 2000, 2001; Malloy and Milling, 2010), gambling (Bouchard et al., 2017), and more (Freeman et al., 2017). Non-traditional uses of VR for psychotherapeutic purposes (e.g., immersive and interactive psychoeducation, skill practicing, task feedback, and more) should also be explored in future research and will likely benefit from being developed in close collaboration with clinicians.

### Strengths and Limitations

Strengths of the current study include a sample of potential VRET adopters and a survey covering both knowledge of, familiarity with, and attitudes toward VRET. Although not designed as a direct replication, survey questions were largely based on past reports (Segal et al., 2011; Schwartzman et al., 2012), allowing cautious inferences about changes in attitudes over time. Limitations of the current study include the use of a cross-sectional design and *ad hoc*, non-exhaustive self-reported measures. Knowledge of VRET and VR technology was self-reported, rather than tested and there were no measures of general attitudes toward and affinity for new technology. Importantly, a longitudinal design would be required to draw more definitive conclusions as to changes in therapists’ attitudes over time. A longitudinal design would also be required to infer whether experience of using VR (in any setting) has a causal effect on attitudes and self-rated likelihood of future use. Second, the sample size was only moderate, although adequate for the statistical methods used and similar to previous work (Segal et al., 2011; Schwartzman et al., 2012), and the data collection procedure did not allow for an estimation of how many conference participants that refused participation. The sample characteristics did, however, not indicate any sample bias. Third, the surveyed list of positive and negative aspects of VRET was not designed to be exhaustive and was not empirically derived, nor could participants supply and rate their own aspects. By design, the survey did not cover structural barriers to clinical implementation such as training requirements and rigidity of existing health care systems (reimbursement models, evidence, and medical technology requirements, etc.), which should be examined in future studies. Fourth, although the conference from which participants were recruited attracted a large proportion of clinician attendees not involved in research, and time spent between research and clinic was not a significant predictor of perceived likelihood of future use, we cannot rule out the possibility that greater variation in attitudes toward VR would have been observed in a non-selected sample. Finally, as made necessary by our intent to survey views and attitudes toward VR treatments also among those totally unfamiliar with this technology, a small minority of participants were given a basic description of VR, which was not standardized and the occurrence of which was not recorded.

## Conclusion

In this first survey of VRET attitudes among practicing CBT clinicians in the era of consumer VR platforms, we show that clinicians overall have a positive attitude toward VRET, in line with past findings. We show for the first time that clinicians view VR as applicable not only in conducting exposure therapy to treat anxiety disorders, but also a range of other disorder types. A high financial cost is no longer viewed as a large barrier to adopting the technology in clinical practice, although there were other concerns, e.g., that treatment improvements would not translate into real-world improvements. Negative attitudes were a larger negative predictor of self-rated likelihood of future use than positive attitudes were a positive predictor, and partially mediated the association between knowledge of VRET and likelihood of future use, suggesting that educational efforts should be aimed at addressing negative concerns rather than promoting positive aspects. We conclude that therapist’s attitudes toward VRET do not appear to constitute a major barrier to implementing the next generation of VR technology in regular clinical practice.

## Author Contributions

PL, AM, LR, and PC designed the study. EZ, AM, PL, and PC collected the data. PL analyzed the data and drafted the manuscript. GA facilitated the study execution and aided in interpretation of findings. PL, AM, EZ, LR, GA, and PC critically reviewed the draft and made significant contributions to the final version.

## Conflict of Interest Statement

Since initiating and designing the study, and collecting data, author PL has received consulting fees from Mimerse, a technology company developing VR applications for clinical use. Mimerse did not fund the current study, nor had any role in the design or execution of the study, analyses or the publication process. The remaining authors declare that the research was conducted in the absence of any commercial or financial relationships that could be construed as a potential conflict of interest. The reviewer LV and handling Editor declared their shared affiliation at the time of the review.
