# Supplementary material for: Attitudes Toward and Familiarity With Virtual Reality Therapy Among Practicing Cognitive Behavior Therapists: A Cross-Sectional Survey Study in the Era of Consumer VR Platforms
Source: Front Psychol. 2019 Feb 8;10:176. doi: 10.3389/fpsyg.2019.00176 (PMC6376952; doi:10.3389/fpsyg.2019.00176)
Supplement: Supplementary file 1 [file Data_Sheet_1.PDF]

Your views on and experience of using Virtual Reality in therapy

We are interested in how cognitive behavior therapists view the potential of using Virtual Reality technology in therapy. Responding to this survey will only take 3-5 minutes. To thank you for your kind participation, you will be given a scratch lottery ticket!

This survey is conducted by members of professor Per Carlbring's research group at Stockholm University. Participation is anonymous and complies with ethical standards. The results of this survey will be published in an academic journal. By submitting your response, you consent to these terms.

*Required

1. **Do you work clinically with cognitive behavior therapy?** *

   Mark only one oval.

   - Yes, I conduct CBT with patients on occasion or regularly
   - No, I do not conduct any CBT (this is not the right survey for you)

2. **What is your sex?** *

   Mark only one oval.

   - Man
   - Woman
   - Other

3. **What is your age (in years)?** *

   ....

4. **What is your training background?** *

   Mark only one oval.

   - Psychologist
   - Counselor
   - Psychiatrist
   - Social worker
   - Nurse
   - Other: ....

5. **How many years have you been practicing?** *

   ....

6. **What type of mental health problems do you primarily work with?** *
   Tick all that apply.

   - [ ] Anxiety disorders
   - [ ] Couples/family therapy
   - [ ] Disruptive behavior disorders
   - [ ] Eating disorders
   - [ ] Gambling disorder
   - [ ] Mood disorders
   - [ ] Neurodevelopmental disorders (ADHD, autism etc.)
   - [ ] Personality disorders
   - [ ] Psychotic disorders
   - [ ] Psychosomatic disorders
   - [ ] Substance dependence and abuse
   - [ ] Other: ....

7. **How do you currently spend your time between clinical work and research/other?** *
   Mark only one oval.

	0	1	2	3	4	5	6	7	8	9	10	
Only research /other	◯	◯	◯	◯	◯	◯	◯	◯	◯	◯	◯	Only clinical

Your views on and experience of using Virtual Reality in therapy

8. **How familiar are you with Virtual Reality technology in general?** *
   Mark only one oval.

	0	1	2	3	4	5	6	7	8	9	10	
Not at all	◯	◯	◯	◯	◯	◯	◯	◯	◯	◯	◯	Very familiar

9. **Are you aware that there are now several consumer VR platforms commercially available, such as Google Cardboard, Samsung Gear, HTC Vive and Oculus Rift?**
   Mark only one oval.

   - ◯ Yes
   - ◯ No

10. **If you were to guess, how much do you think a high-performance, smartphone-based VR headset (such as the Samsung Gear VR) costs? (In US dollars)** *

   ....

11. **How familiar are you with the research on the efficacy of VR exposure therapy? ***
Mark only one oval.

	0	1	2	3	4	5	6	7	8	9	10	
Not at all	◯	◯	◯	◯	◯	◯	◯	◯	◯	◯	◯	Very familiar

12. **Have you ever used Virtual Reality in a clinical setting? ***
Mark only one oval.

- ◯ No
- ◯ Yes, on occasion
- ◯ Yes, frequently

13. **Have you ever used Virtual Reality in any other setting? (E.g. gaming)**
Mark only one oval.

- ◯ No
- ◯ Yes, on occasion
- ◯ Yes, frequently

14. **How likely do you think it is that you will start using VR clinically within the next two years? ***
Mark only one oval.

	0	1	2	3	4	5	6	7	8	9	10	
Not at all	◯	◯	◯	◯	◯	◯	◯	◯	◯	◯	◯	Definitively

15. **With which types of mental health problems do you think VR can be used? ***
Tick all that apply.

- ☐ Anxiety disorders
- ☐ Couples/family therapy
- ☐ Disruptive behavior disorders
- ☐ Eating disorders
- ☐ Gambling disorder
- ☐ Mood disorders
- ☐ Neurodevelopmental disorders (ADHD, autism etc.)
- ☐ Personality disorders
- ☐ Psychotic disorders
- ☐ Psychosomatic disorders
- ☐ Substance dependence and abuse
- ☐ Other: ....

16. **These are some possible POSITIVE potentials of VR exposure therapy. Please rate how positive you view them** *
Mark only one oval per row.

	0. Matters not	1	2	3	4	5. Very POSITIVE
Exposing clients to objects, situations and tasks that could only be accomplished in a virtual setting	( )	( )	( )	( )	( )	( )
To always have exposure material at hand without preparation	( )	( )	( )	( )	( )	( )
To be able to precisely control and tailor exposure stimuli	( )	( )	( )	( )	( )	( )
To be able precisely control and tailor exposure task/design	( )	( )	( )	( )	( )	( )
Increasing clients' sense of control	( )	( )	( )	( )	( )	( )
Reducing the need to conduct embarassing exposure tasks in public	( )	( )	( )	( )	( )	( )
Reducing session duration or number of sessions	( )	( )	( )	( )	( )	( )
Improving homework exposure tasks in-between sessions	( )	( )	( )	( )	( )	( )
Making exposure less stressful using e.g. gamification	( )	( )	( )	( )	( )	( )

17. **These are some possible NEGATIVE aspects of VR exposure therapy. Please rate how negative you view them** *
Mark only one oval per row.

	0. No concern	1	2	3	4	5. Very NEGATIVE
Cost in acquiring and upgrading the VR equipment	( )	( )	( )	( )	( )	( )
Technical difficulties in operating the VR equipment	( )	( )	( )	( )	( )	( )
Poor quality of the software	( )	( )	( )	( )	( )	( )
Poor quality of the hardware	( )	( )	( )	( )	( )	( )
Ethical or legal concerns	( )	( )	( )	( )	( )	( )
Risk of sensitive data leaks	( )	( )	( )	( )	( )	( )
Reluctance among certain patients to use new technology	( )	( )	( )	( )	( )	( )
Patients unable to grasp and use the technology on their own	( )	( )	( )	( )	( )	( )
Side effects of use such as nausea	( )	( )	( )	( )	( )	( )
Patients not experiencing the VR environment as real enough	( )	( )	( )	( )	( )	( )
Patients experiencing the VR environment as too real	( )	( )	( )	( )	( )	( )
Treatment improvements not translating into real-world improvements	( )	( )	( )	( )	( )	( )

18. **Finally, is there anything you would like to add?**

....

Powered by
Google Forms
